# Chronic alcohol exposure induces hepatocyte damage by inducing oxidative stress, SATB2 and stem cell‐like characteristics, and activating lipogenesis

**DOI:** 10.1111/jcmm.17235

**Published:** 2022-02-13

**Authors:** Wei Yu, Yiming Ma, Sushant K. Shrivastava, Rakesh K. Srivastava, Sharmila Shankar

**Affiliations:** ^1^ Kansas City VA Medical Center Kansas City Missouri USA; ^2^ Department of Pharmaceutics Indian Institute of Technology Banaras Hindu University Varanasi U.P. India; ^3^ Department of Genetics Louisiana State University Health Sciences Center New Orleans Louisina USA; ^4^ Stanley S. Scott Cancer Center Department of Genetics Louisiana State University Health Sciences Center New Orleans Louisina USA; ^5^ A.B. Freeman School of Business Tulane University New Orleans Louisina USA; ^6^ John W. Deming Department of Medicine Tulane University School of Medicine New Orleans Louisina USA; ^7^ Southeast Louisiana Veterans Health Care System New Orleans Louisina USA

**Keywords:** alcohol, cancer stem cell, hepatocellular carcinoma, pluripotency, SATB2, steatosis, Wnt

## Abstract

Alcohol is a risk factor for hepatocellular carcinoma (HCC). However, the molecular mechanism by which chronic alcohol consumption contributes to HCC is not well understood. The purpose of the study was to demonstrate the effects of chronic ethanol exposure on the damage of human normal hepatocytes. Our data showed that chronic exposure of hepatocytes with ethanol induced changes similar to transformed hepatocytes that is, exhibited colonies and anchorage‐independent growth. These damaged hepatocytes contained high levels of reactive oxygen species (ROS) and showed induction of the SATB2 gene. Furthermore, damaged hepatocytes gained the phenotypes of CSCs which expressed stem cell markers (CD133, CD44, CD90, EpCAM, AFP and LGR5), and pluripotency maintaining factors (Sox‐2, POU5F1/Oct4 and KLF‐4). Ethanol exposure also induced Nanog, a pluripotency maintaining transcription factor that functions in concert with Oct4 and SOX‐2. Furthermore, ethanol induced expression of EMT‐related transcription factors (Snail, Slug and Zeb1), N‐Cadherin, and inhibited E‐cadherin expression in damaged hepatocytes. Ethanol enhanced recruitment of SATB2 to promoters of Bcl‐2, Nanog, c‐Myc, Klf4 and Oct4. Ethanol also induced activation of the Wnt/TCF‐LEF1 pathway and its targets (Bcl‐2, Cyclin D1, AXIN2 and Myc). Finally, ethanol induced hepatocellular steatosis, SREBP1 transcription, and modulated the expression of SREBP1c, ACAC, ACLY, FASN, IL‐1β, IL‐6, TNF‐α, GPC3, FLNB and p53. These data suggest that chronic alcohol consumption may contribute towards the development of HCC by damaging normal hepatocytes with the generation of inflammatory environment, induction of SATB2, stem cell‐like characteristics, and cellular steatosis.

## 1 INTRODUCTION

Hepatocellular carcinoma (HCC) is one of the primary liver cancers, predicted to be the sixth most commonly diagnosed cancer and the third leading cause of cancer death worldwide.^1^ The worldwide HCC incidence is 10.1 cases per 100,000 person‐years.^1^ The burden of HCC in 2012 was 14 million and is expected to rise to 22 million in the next two decades.^2^ The incidence of HCC in the US has tripled over the last four decades. HCC has an average five‐year survival of <20%.^2^ In the United States, approximately 42,230 adults are expected to be diagnosed with liver cancer, and 30,230 deaths are expected to occur from this disease in 2021.^2^

The development of HCC is complex.^3^ It involves sustained inflammatory damage leading to hepatocyte necrosis, regeneration and fibrotic deposition. Epidemiological data strongly suggest that heavy drinking increases the risk for liver cancer.^4^, ^5^, ^6^ Alcohol consumption is an independent risk factor and a primary cause of HCC.^7^, ^8^, ^9^, ^10^ According to the National Institute on Alcohol Abuse and Alcoholism (NIAAA), a standard alcoholic drink in the United States contains 14.0 g (0.6 ounces) of pure alcohol. Heavy alcohol drinking is defined as the consumption of more than three drinks on any day or more than seven drinks per week for women, and more than four drinks on any day or more than 14 drinks per week for men. As per the recent report from the National Cancer Institutes (USA), the chances of getting liver cancer increase significantly with five or more drinks per day. However, the molecular mechanism(s) by which ethanol (EtOH) induces hepatocyte damage/malignant transformation leading to HCC development is not well understood.

SATB2 (special AT‐rich binding protein‐2) gene is required for normal mammalian development;^11^, ^12^ however, it is not expressed in normal adult tissues, including normal hepatocytes.^13^, ^14^, ^15^ SATB2 is a DNA binding protein that specifically binds nuclear matric attachment regions and is involved in transcription regulation and chromatin remodelling,^16^ and thus, regulates gene expression.^17^, ^18^, ^19^ *SATB2* ^−/−^ mice are defective in bone development and osteoblast differentiation.^19^ In addition, *satb2* ^−/−^ mice and humans with loss‐of‐function *satb2* mutations develop craniofacial abnormalities, including orofacial clefting.^19^, ^20^, ^21^, ^22^ SATB2 is essential for proper facial patterning of the embryo and normal bone development.^19^ These defects have been attributed to an increased expression of specific members of the *Hox* gene (a subset of homeobox genes) clusters and a decreased expression of osteoblast‐specific genes, whereby satb2 was shown to regulate these genes at the chromatin level.^19^ SATB2 regulates the transcription of those genes, which modulates pluripotency maintaining factors, cell growth and stemness.^11^, ^23^, ^24^, ^25^, ^26^, ^27^ Inappropriate activation of this SATB2 gene may be the cause of malignant cellular transformation.^14^, ^15^, ^28^ Generation of stem cells/progenitor cells during cellular transformation is the primary cause of cancer initiation, promotion and metastasis.^29^, ^30^, ^31^, ^32^ We have recently demonstrated that chronic exposure of pancreatic ductal epithelial cells to ethanol induces transformation, which causes cells to gain the functions of cancer stem cells (CSCs). SATB2 can directly activate Wnt/β‐catenin/TCF‐LEF pathway, which regulates stem cell self‐renewal and transformation.^28^ Our recent data demonstrate that inhibition of SATB2 expression by Crisp/Cas9 technique suppresses epithelial–mesenchymal transition, stem cell markers and pluripotency maintaining factors in CSCs derived from HCC.^13^ Liver CSCs are resistant to chemotherapy and radiotherapy.^33^ Several markers of liver CSCs such as epithelial cellular adhesion molecule (EpCAM, CD326), CD90, CD44, CD24, CD133 and AFP, either alone or in combination, have been used.^32^, ^34^ Moreover, the role of SATB2 in ethanol‐induced transformation/damage of hepatocytes is unknown.

The main goal of this paper is to examine the molecular mechanisms by which EtOH induces damage to human normal hepatocytes (i.e. malignant transformation). Our data have demonstrated that chronic exposure of human normal hepatocytes to EtOH induces an inflammatory environment, SATB2 expression and activates Wnt3a/β‐catenin/TCF‐LEF pathway. Furthermore, during ethanol‐induced hepatocyte damage, hepatocytes gained the phenotypes of progenitor cells / CSCs and expressed pluripotency maintaining factors and EMT markers. In addition, ethanol‐induced hepatocellular steatosis and SREBP1 transcription and also modulated the expression of sterol‐regulatory element binding protein 1 (SREBP1), acetyl‐Co‐A Carboxylase (ACAC), ATP citrate lyase (ACLY), fatty acid synthase (FASN), interleukin‐1β (IL‐1β), interleukin‐6 (IL‐6), tumour necrosis factor‐ α (TNF‐α), glypican 3 (GPC3), filamin B (FLNB), and tumour suppressor protein 53 (p53). These data suggest that chronic alcohol consumption may contribute to the development of HCC by damaging normal hepatocytes by creating an inflammatory environment, induction of SATB2, stemness and cellular steatosis.

## 2 MATERIALS AND METHODS

### 2.1 Reagents

HEK293T cells were purchased from American Type Culture Collection (ATCC), Manassas, VA. A set of Crisper/cas9 lentiviral plasmids against human SATB2 gene, and non‐targeting controls were purchased from GeneCopoeia^TM^ (Rockville, MD). BD Matrigel^TM^ was purchased from BD Bioscience (San Jose, CA). TRIzol was purchased from Invitrogen (Grand Island, NY). 8‐hydroxy 2 deoxyguanosine ELISA (8‐OHdG) was obtained from Abcam (Waltham, MA). PEG‐it virus precipitation solution was purchased from SBI System Biosciences (Palo Alto, CA). Dulbecco's Modified Eagle's Medium (DMEM), foetal bovine serum (FBS), antibiotics were purchased from Thermo Fisher Scientific (Waltham, MA).

CellTiter‐Glo^®^ Luminescent Cell Viability Assay and Luciferase assay kits were purchased from Promega Corporation (Madison, WIWI). All other chemicals were purchased from Sigma‐Aldrich (St. Louis, MO).

### 2.2 Cell culture

293T cells were grown in Dulbecco's Modified Eagle's Medium (DMEM) with antibiotics with 10% Foetal Bovine Serum (HyClone). EpCAM^+^/CD44^+^/CD133^+^ human liver CSCs were isolated from primary hepatocellular carcinoma (obtained from Celprogen, Torrance, CA) and grown in a well‐defined stem cell culture medium as per the supplier's instructions. All cells were checked for the absence of *Mycoplasma* using the kit (Lonza).

### 2.3 Lentiviral particle production and transduction

The protocol for lentivirus production and transduction have been described elsewhere.^35^ Briefly, 293T cells were transfected with four µg of plasmid and four µg of the lentiviral vectors using Lipofectamine‐3000 according to the manufacturer's protocol (Invitrogen). PEG‐it virus precipitation solution (SBI System Biosciences) was added to the supernatant, and ultracentrifugation was performed to collect concentrated viral particles. Hepatocytes were transduced with lentiviral particles with 6 μg/ml polybrene (Invitrogen).

### 2.4 Colony formation assay

For colony formation assays, hepatocytes were grown in matrigel‐coated plates and treated with or without ethanol (100 mm). The culture medium was replaced with fresh medium every three days, and cells were treated with ethanol. The hepatocytes were treated for a total of two weeks. After incubation, colonies were fixed with methanol, stained with 0.5% crystal violet and visualized under a microscope.

### 2.5 8‐hydroxy 2 deoxyguanosine ELISA Kit (8‐OHdG) ELISA

8‐hydroxy 2 deoxyguanosine (8‐OHdG) ELISA kit was used for the quantification of 8‐OHdG as per the manufacturer's instructions (Abcam). The ELISA detects 8‐OHdG with an assay range of 0.94–60 ng/mL, with a 0.59 ng/ml sensitivity.

### 2.6 Oil Red O staining

To examine fat accumulation, the hepatocytes were rinsed with cold phosphate‐buffered saline (PBS) and fixed in 10% paraformaldehyde for 30 min. After the hepatocytes were washed with 60% isopropanol, they were stained for at least 1 h in a freshly diluted Oil Red O solution (six parts Oil Red O stock solution and four parts H_2_O; 0.5% Oil Red O Stock solution in isopropanol). After removing the excess stain from cells, they were washed twice with 60% isopropanol. Lipid droplets appear red under the microscope. The stained lipid droplets were then extracted with isopropanol for quantification by measuring their absorbance at 490 nm. 100% isopropanol was used as background control.

### 2.7 Chromatin Immunoprecipitation (ChIP) assay

Pancreatic CSCs were fixed with 1% formaldehyde for 15 min (RT), quenched with 125 mM glycine for 5 min (RT), centrifuged and resuspended in RIPA buffer containing protease inhibitors, and incubated on ice (10 min). Samples were sonicated (Heat Systems‐Ultrasonic device) to shear chromatin to an average length of about 1 Kb, transferred to 1.5 ml tubes, and microcentrifuged for 10 min (6000 g). Supernatants were collected in 1.5 ml tubes containing 1 ml of the Dilution Buffer (0.01% SDS, 1.1% Triton, 1.2 mM EDTA, 167 mM NaCl, 17 mM Tris, pH 8). 3 μg of SATB2 antibody was added to the samples. The mixtures were incubated overnight at 4°C, 5 μl of protein‐A and protein‐G magnetic beads (Invitrogen) were added for two h. Beads were collected with a magnet (Thermo), washed 4× with 1 ml of each of four Wash Buffers (Wash Buffer 1: 0.1% SDS, 1% Triton, 2 mM EDTA, 150 mM NaCl, 20 mM Tris, pH 8; Wash Buffer 2: 0.1% SDS, 1% Triton, 2 mM EDTA, 500 mM NaCl, 20 mM Tris, pH 8; Wash Buffer 3: 0.25 M LiCl, 1% NP‐40, 1% deoxycholate, 1 mM EDTA, 10 mM Tris, pH 8; Wash Buffer 4:10 mM Tris, pH 8, 1 mM EDTA). After the last wash, 50 µl of a 10% Chelex‐100 (Bio‐Rad) resin solution was added to the beads, samples were boiled (10 min) in a heat block, microcentrifuged (1 min, 6000 g), supernatants followed by the addition of 50 μl of MQ water back to the beads, microcentrifuged again (1 min, 6000 g), and the new supernatant pooled with the previous one. 1–3 µl elutions were used for PCR reactions.

### 2.8 Lentiviral particle production and transduction

The protocol for lentivirus production and transduction have been described elsewhere.^36^, ^37^ In brief, lentivirus was produced by triple transfection of HEK 293T cells. Packaging 293T cells were plated in 10‐cm plates at a cell density of 5 × 10^6^ a day before transfection in DMEM containing 10% heat‐inactivated foetal bovine serum. 293T cells were transfected with four µg of plasmid and four µg of the lentiviral vector using lipid transfection (Lipofectamine‐2000/Plus reagent, Invitrogen) manufacturer's protocol. Viral supernatants were collected and concentrated by adding PEG‐it virus precipitation solution (System Biosciences Inc, SBI) to produce virus stocks with titres of 1 × 10^8^ to 1 × 10^9^ infectious units per ml. Viral supernatant was collected for three days by ultracentrifugation and concentrated 100‐fold. Titres were determined on 293T cells. Cells were transduced with lentiviral particles expressing the gene of interest.

### 2.9 Quantitative real‐time PCR

Total RNA was extracted with TRIzol reagent (Invitrogen), and the cDNA was generated by the Reverse Transcription System (Promega) in a 20 μl reaction containing 1 μg of total RNA. A 0.5 μl aliquot of cDNA was amplified by Fast SYBR Green PCR Master Mix (Applied Biosystems) in each 20 μl reaction. PCR reactions were run on the ABI 7900 Fast Real‐Time PCR system (Applied Biosystems).

### 2.10 TCF/LEF reporter assay

Lentiviral particles expressing cop‐GFP and luciferase genes (TCF/LEF‐mCMV‐EF1‐Neo) were prepared as described elsewhere.^38^ Cells were transduced with lentiviral particles. Transduced cells (5–10,000 cells per well) were seeded in 96‐well plates for 48 h. At the end of the incubation period, luciferase reporter activity was measured per the manufacturer's instructions (Promega Corp., Madison, WI).

### 2.11 Statistical analysis

All analyses were performed using GraphPad Prism Software (GrafPad Software, Inc., San Diego, CA). Statistical differences between groups were analysed using the Student *t*‐test or Analysis of Variance (ANOVA). Significant differences among groups were calculated at *p* < 0.05. The mean ± SD or SE was calculated for each experimental group.

## 3 RESULTS

### 3.1 Ethanol induces hepatocyte damage by generating reactive oxygen species and SATB2 expressions

**FIGURE 1:**
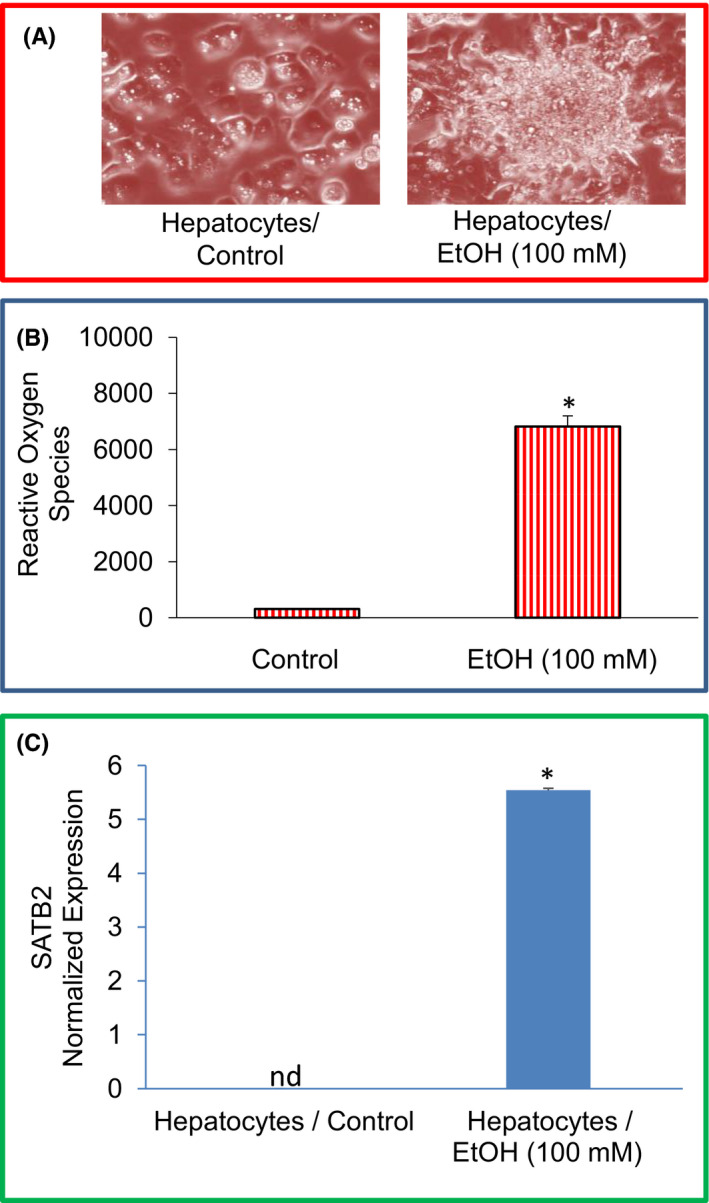
Ethanol induces hepatocytes damage by generating reactive oxygen species and inducing SATB2 expression. (A), Morphological changes in human hepatocytes. Phase‐contrast imaging of Hepatocytes/Control and EtOH‐treated hepatocytes (Hepatocytes/Ethanol). Hepatocytes were cultured for two weeks with ethanol (100 mM). Photographs were taken under a phase‐contrast microscope. (B), Generation of reactive oxygen species. Hepatocytes were treated with or without ethanol for two weeks. Reactive oxygen species were measured as per the manufacturer's instructions. (C), SATB2 expression by qRT‐PCR in Hepatocytes/Control and Hepatocytes/Ethanol transformed cells. qRT‐PCR data represent mean ± SD (*n* = 4). *Significantly different from control, *p* < 0.05

We first examined the effects of ethanol on the morphological changes of human normal hepatocytes. Human normal hepatocytes were grown on the matrigel‐coated dishes with a cell culture medium in the presence or absence of EtOH (100 mM) for two weeks (Figure 1A). Exposure of hepatocytes to ethanol induced cellular transformation as evident by the formation of colony‐like structures, loss of contact inhibition and disoriented growth (Figure 1A).

High concentrations of ethanol cause the production and release of many free radicals and inflammatory mediators, which leads to liver injury. We, therefore, examine the effects of ethanol on ROS production in hepatocytes (Figure 1B). Compared to the untreated control group, chronic exposure of human normal hepatocytes with ethanol‐induced ROS. These data suggest that ROS production during ethanol exposure of hepatocytes may be responsible for cellular damage.

It has been reported that the SATB2 plays an essential role in malignant transformation and stemness. We, therefore, examined the mechanism of ethanol‐induced transformation of normal hepatocytes by comparing the expression of SATB2 in untreated hepatocytes and ethanol‐transformed hepatocytes (Hepatocytes/Ethanol). As shown in Figure 1C, exposure of normal hepatocytes to ethanol (100 nM) resulted in the induction of the SATB2 gene.

### 3.2 Ethanol‐transformed hepatocytes express stem cell markers and pluripotency maintaining factors

**FIGURE 2:**
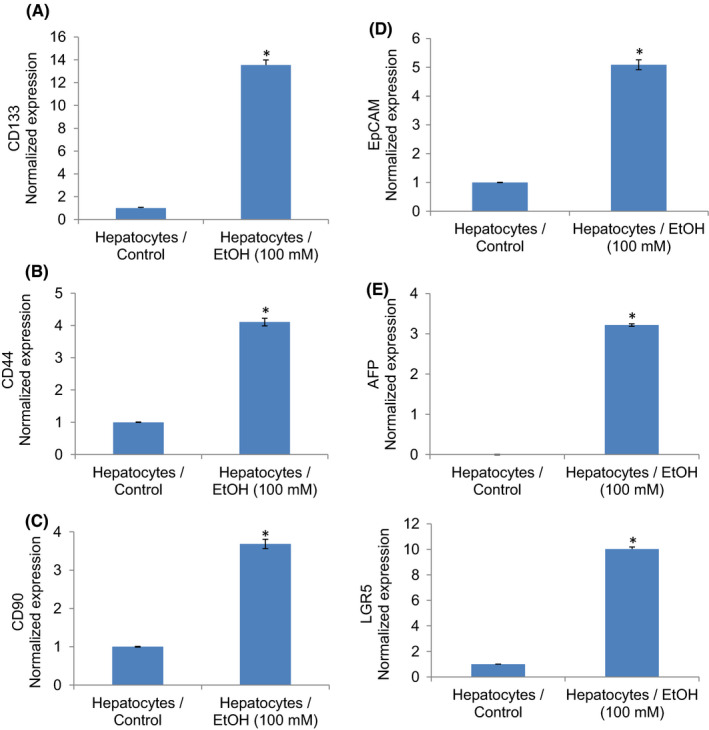
Ethanol induces stem cell markers and AFP in human hepatocytes. (A–E), Expression of stem cell markers. Normal human hepatocytes were treated with or without ethanol. RNA was extracted from cells, and qRT‐PCR analysis was performed to measure the expression of CD133, CD44, CD90, EpCAP, AFP and LGR5. GAPDH was used as an internal control. Data represent mean ± SD (*n* = 4). *Significantly different Hepatocytes/Control, *p* < 0.05. Note that the expression of genes in the control group was normalized to 1

**FIGURE 3:**
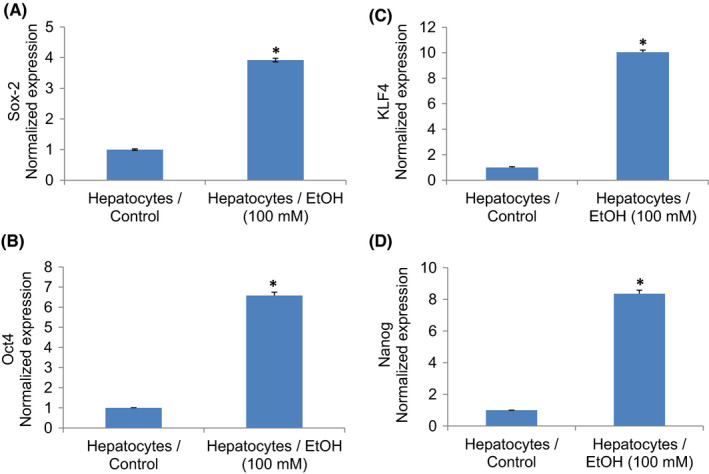
Chronic ethanol exposure of human hepatocytes induces pluripotency maintaining factors. (A–D), Expression of pluripotency maintaining factors. RNA was extracted, and qRT‐PCR analysis was performed to measure the expression of Sox‐2, Oct4, KLF4 and Nanog, as we described elsewhere.^40^, ^65^ GAPDH was used as an internal control. Data represent mean ± SD (*n* = 4). *Significantly different Hepatocytes/Control, *p* < 0.05. Note that the expression of genes in the control group was normalized to 1

We next examined whether ethanol**‐**transformed hepatocytes gained the phenotypes of CSCs by measuring stem cell markers (Figure 2) and pluripotency maintaining factors (Figure 3). Ethanol‐transformed hepatocytes expressed significantly higher stem cell markers (CD24, CD44, and CD133) and pluripotency maintaining factors (Oct4 and Sox‐2) than control hepatocytes. Exposure of hepatocytes to ethanol also induced Nanog, a pluripotency maintaining transcription factor that functions in concert with Oct4 and SOX‐2. These data suggest that ethanol can induce hepatocytes’ transformation, and these transformed cells gained the phenotypes of CSCs.

### 3.3 Chronic exposure of hepatocytes to ethanol induces markers of epithelial to mesenchymal transition (EMT)

**FIGURE 4:**
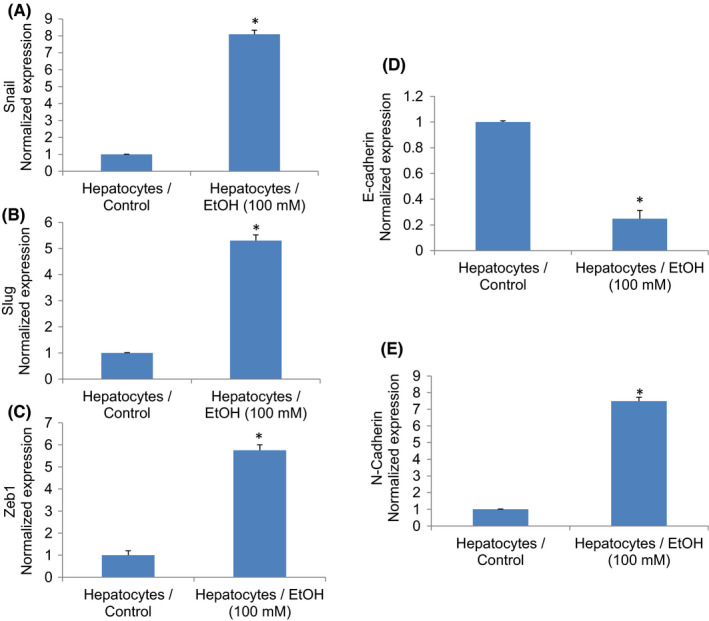
Chronic exposure of human normal hepatocytes modulated expression of EMT‐related genes. (A–C), Expression of EMT‐related transcription factors. Human normal hepatocytes were treated with ethanol (100 mM) for two weeks. RNA was extracted, and qRT‐PCR analysis was performed to measure the expression of Snail, Slug and Zeb1. GAPDH was used as an internal control. Data represent mean ± SD (*n* = 4). *Significantly different from Hepatocytes/Control cells, *p* < 0.05. (D–E), Expression of cadherins. Human normal hepatocytes were treated with ethanol (100 mM) for two weeks. RNA was extracted, and qRT‐PCR analysis was performed to measure E‐cadherin and N‐cadherin. GAPDH was used as an internal control. Data represent mean ± SD (*n* = 4). *Significantly different from Hepatocytes/Control, *p* < 0.05

The induction of epithelial to mesenchymal transition (EMT) is one of the characteristics of CSCs. Conversion of a cell from an epithelial to mesenchymal transition leads to increased migratory and invasive properties, and thus, facilitates the initiation of metastasis.^38^, ^39^ Transcription factors Snail, Slug and Zeb1 are induced in cells undergoing EMT. Ethanol induces the expression of Snail, Slug and Zeb1 in hepatocytes (Figure 4A–C). During EMT, the expression of E‐cadherin is inhibited, and the expressions of N‐cadherin and vimentin are induced.^39^, ^40^ We, therefore, measured the expression of E‐cadherin, N‐cadherin and vimentin in control and EtOH‐transformed cells. Ethanol treatment inhibited the expression of E‐cadherin and upregulated the expression of N‐cadherin and vimentin in Hepatocytes/Ethanol cells (Figure 4D,E). These data suggest that exposure of hepatocytes to EtOH can induce EMT by regulating genes involved in EMT.

### 3.4 Ethanol‐induced SATB2 binds to promoters of Bcl‐2, Nanog, c‐Myc, KLF4 and Oct4

**FIGURE 5:**
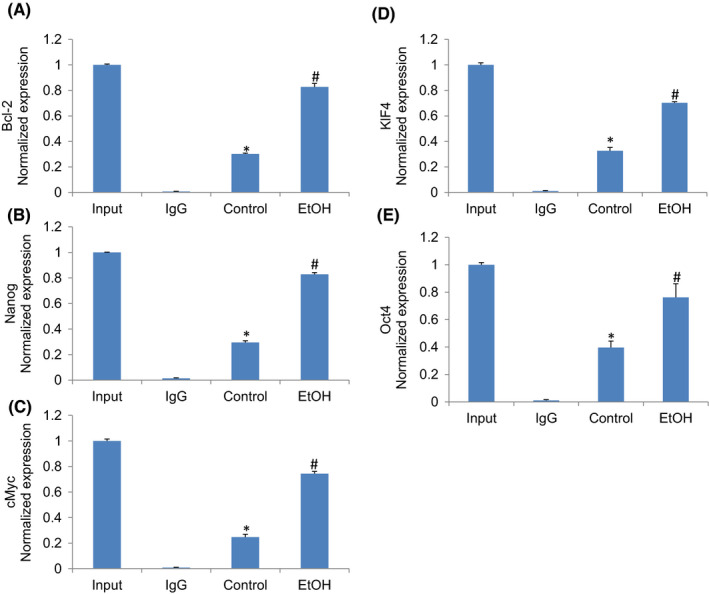
Binding of SATB2 to promoters of Bcl‐2, Bsp, Nanog, c‐Myc, XIAP, Klf4 and Hoxa2. Nuclear extracts were prepared from pancreatic CSCs. Chromatin immunoprecipitation (ChIP) assays followed by qRT‐PCR were performed to examine the binding of the SATB2 to the promoters of Bcl‐2, Nanog, c‐Myc, Klf4 and Oct4. Data represent mean ± SD (*n* = 4). *Significantly different from Hepatocytes/Control, *p* < 0.05

Chromatin immunoprecipitation (ChIP) assays are performed to identify genome regions with which DNA‐binding proteins, such as transcription factors, associate. In ChIP assays, proteins bound to DNA are temporarily crosslinked, and the DNA is sheared before cell lysis. The target proteins are immunoprecipitated along with the crosslinked nucleotide sequences, and the DNA is then removed and identified by PCR. Next, we performed a ChIP assay to examine whether SATB2 can occupy the promoters of genes that regulate cell proliferation (Bcl‐2), pluripotency and self‐renewal (Nanog, c‐Myc, KLF4, and Oct4). Hepatocytes were treated with ethanol (100 mM) for two weeks, and a ChIP assay was performed using the SATB2 antibody. As shown in Figure 5, SATB2 could bind to promoters of Bcl‐2, Nanog, Myc, KLF4 and Oct4. These data suggest that SATB2 can directly regulate the expression of Bcl‐2, Nanog, Myc, KLF4 and Oct4.

### 3.5 Chronic exposure of human hepatocytes to ethanol regulates Wnt/β‐catenin/TCF/LEF pathway and its downstream targets Bcl‐2, Cyclin D1, AXIN2 and c‐Myc

**FIGURE 6:**
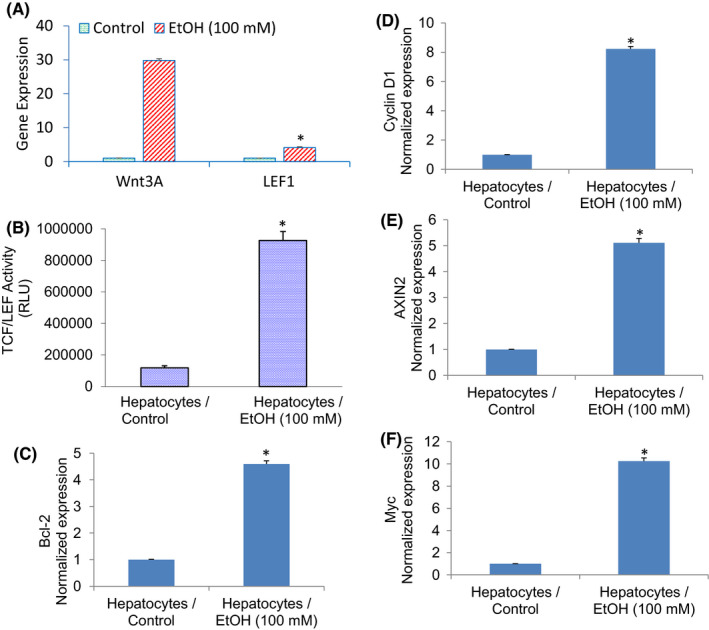
Ethanol regulates the Wnt/TCF‐LEF pathway and the expression of its target genes Bcl‐2, Cyclin D1, AXIN2 and Myc. (A), Expression of Wnt3A and LEF1 expression. Human normal hepatocytes were treated with or without ethanol (100 mM) for two weeks. RNA was extracted, and qRT‐PCR analysis was performed to measure the expression of Wnt3A and LEF1. GAPDH was used as an internal control. Data represent mean ± SD (*n* = 4). *Significantly different from control, *p* < 0.05. (B), TCF/LEF1 Activity. Hepatocytes were transduced with lentiviral particles expressing TCF‐LEF luciferase construct and exposed to ethanol. TCF/LEF activity was measured by luciferase assay. Data represent mean (*n* = 4) ± SD. *Significantly different from scrambled control group (*p* < 0.05). (C–F), Expression of Bcl‐2, Cyclin D1, AXIN2 and Myc expression. Human normal hepatocytes were treated with or without ethanol (100 mM) for two weeks. RNA was extracted, and qRT‐PCR analysis was performed to measure the expression of Bcl‐2, Cyclin D1, AXIN2 and Myc. GAPDH was used as an internal control. Data represent mean ± SD (*n* = 4). *Significantly different from control, *p* < 0.05

Wnt/β‐catenin/TCF/LEF pathway regulates malignant transformation and plays a significant role in HCC development. The nuclear translocation of β‐catenin is assumed to play a crucial role in the malignant transformation of liver progenitor cells.^41^ Since the Wnt3a promoter contains SATB2 binding sites, we measured the expression of Wnt3a and the transcriptional activity of TCF/LEF1. Ethanol induced the expression of Wnt3a and LEF1 (Figure 6A). We next examined transcriptional activation of TCF/LEF1 by luciferase assay in hepatocytes treated with or without ethanol (Figure 6B). Hepatocytes exposed to ethanol showed enhanced TCF/LEF1 activity.

Since ethanol induced TCF/LEF1 activity in hepatocytes, we sought to examine whether ethanol can induce TCF/LEF target gens such as Bcl‐2, Cyclin d1, AXIN2 and Myc. As shown in Figure 6D–F, chronic exposure of hepatocytes to ethanol induced the expression of Bcl‐2, Cyclin D1, AXIN2 and Myc. These data suggest that ethanol can induce molecular changes in hepatocytes by activating Wnt/β‐catenin/TCF/LEF1 pathway.

### 3.6 Ethanol induces hepatocellular steatosis, induces SREBP1 target gens, induces inflammation and causes oxidative stress

**FIGURE 7:**
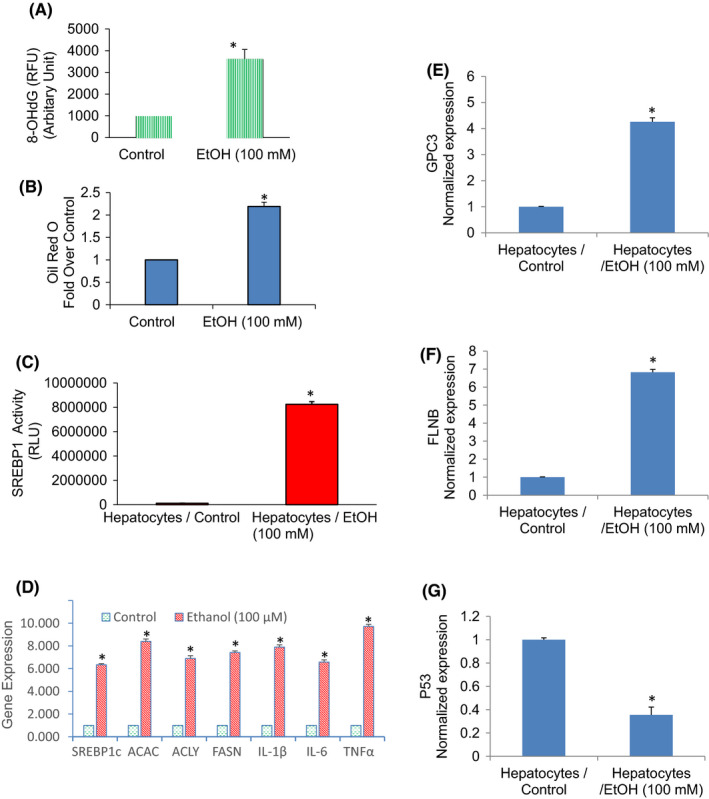
Induction of hepatic steatosis by ethanol. (A), Human hepatocytes were treated with ethanol (100 mM) for two weeks. The quantification of 8‐hydroxy two deoxyguanosine (8‐OHdG) was performed by ELISA as per the manufacturer's instructions (Abcam). (B), Human hepatocytes were treated with ethanol (100 mM) for two weeks. Cells were harvested, fixed and stained with Oil Red O solution. The stained lipid droplets were then extracted for quantification by measuring their absorbance at 490 nm. (C), SREBP1 transcription activity. Hepatocytes were transduced with lentiviral particles harbouring SREBP1 luciferase construct and exposed to ethanol. SREBP1 activity was measured by luciferase assay. Data represent mean (*n* = 4) ± SD. *Significantly different from scrambled control group (*p* < 0.05). (D), Hepatocytes were treated with ethanol (100 mM) for two weeks, and the expression of SREBP1, ACAC, ACLY, FASN, IL‐1β, IL‐6 and TNFα was measured by qRT‐PCR. Data represent mean ± SD (*n* = 4). *Significantly different from Control, *p* < 0.05. (E–G), Hepatocytes were treated with ethanol (100 mM) for two weeks, and the expression of GPC3, FLNB and p53 was measured by qRT‐PCR. Data represent mean ± SD (*n* = 4). *Significantly different from Control, *p* < 0.05

Oxidative stress is involved in the pathophysiology of many diseases. 8‐hydroxy‐2′‐deoxyguanosine (8‐OHdG) is considered the major type of DNA damage and is the commonly used biomarker to evaluate cellular oxidative stress.^42^ We, therefore, measured the formation of 8‐OHdG in hepatocytes treated with or without ethanol (Figure 7A). Chronic exposure of hepatocytes produced a significantly higher quantity of 8‐OHdG than an untreated control group.

We next examined the effects of ethanol on lipid accumulation. The lipid accumulation (cellular steatosis) was measured by Oil Red O staining. Chronic exposure of hepatocytes with ethanol resulted in the accumulation of lipid droplets which was evident by Oil Red O staining (Figure 7B). Sterol regulatory element‐binding protein 1 (SREBP1) activates fatty acid and triglyceride metabolism genes. We next examined the effects of ethanol on the transcriptional activation of SREBP1. Chronic exposure of hepatocytes with ethanol resulted in significantly higher transcriptional activity of SREBP1 compared to untreated control (Figure 7C). We next examined the effects of ethanol on the expression of transcription factor (SREBP1c), lipogenic genes (acetyl Co‐A Carboxylase and FASN), and inflammatory cytokines (IL‐6, IL‐1β, TNFα) by q‐RT‐PCR (Figure 7D). Ethanol induces the expression of SREBP1, ACAC, ACLY, FASN, IL‐1β, IL‐6 and TNF‐α in normal hepatocytes. These data suggest that ethanol induces lipogenesis by modulating the transcription of SREBP1 and lipogenic genes and upregulating inflammatory cytokines.

GPC3 encodes for glypican three proteins involved in numerous cell functions, including cell growth, cell proliferation and cell survival. We, therefore, examined the effects of ethanol on the expression of GPC3 in hepatocytes. Chronic exposure of hepatocyte with ethanol induces GPC3 (Figure 7E). Filamin B (FLNB), an actin‐binding protein that provides crucial scaffolds for cell motility and signalling, has also been identified as an RNA‐binding protein. It regulates the expression of those genes which play roles in apoptosis, tumorigenesis, metastases, transmembrane transport and cartilage development. We, therefore, examine the effects of ethanol on the expression of FLNB in human hepatocytes. Chronic exposure of normal hepatocytes with an ethanol induced the expression of the FLNB gene (Figure 7F). The p53 protein mediates tumour‐suppressive functions resulting in either cell death or the maintenance of cell homeostasis.^43^, ^44^ We, therefore, examined the effects of ethanol on 53 expressions on hepatocytes. Chronic exposure of normal hepatocytes with ethanol inhibited the expression of p53 (Figure 7G). These data suggest that chronic exposure of normal hepatocytes with ethanol may disrupt normal homeostasis resulting in altered expression of genes that regulate various cellular functions.

### 3.7 *N*‐acetylcysteine (NAC) attenuates the effects of chronic exposure to ethanol in hepatocytes

**FIGURE 8:**
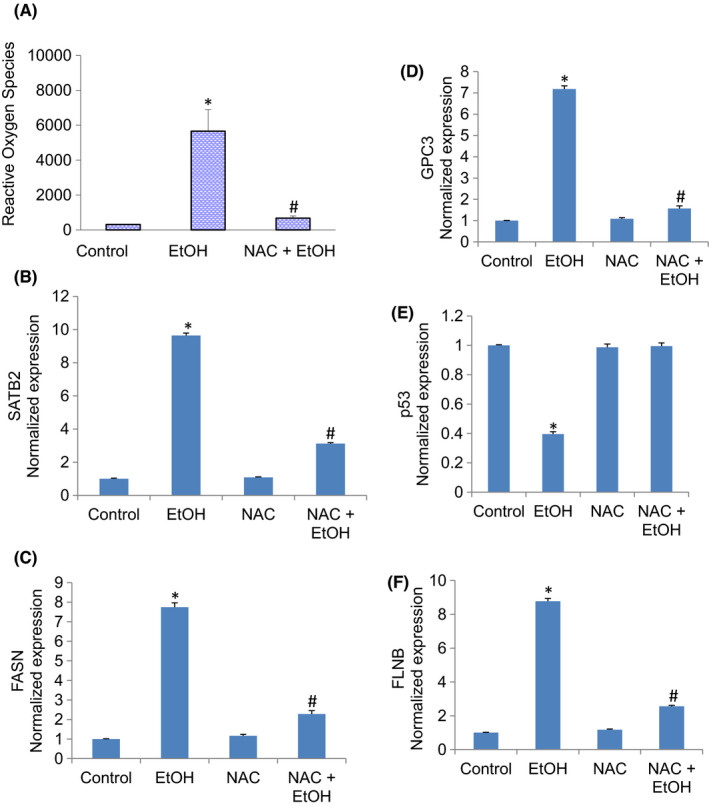
N‐Acetylcysteine (NAC) attenuates the effects of ethanol on ROS production and gene expression. (A), ROS production. Hepatocytes were pretreated by NAC (1 mM) for 30 min, followed by ethanol (100 mM) treatment for two weeks. Production of ROS in hepatocytes was measured as per the manufacturer's instructions. Data represent mean ± SD (*n* = 4). *Significantly different from Control, *p* < 0.05. (B–F), Gene expression. Hepatocytes were pretreated by NAC (1 mM) for 30 min, followed by ethanol (100 mM) treatment for 2 weeks. The expression of SATB2, FASN, GPC3, p53 and FLNB was measured by qRT‐PCR. Data represent mean ± SD (*n* = 4). *Significantly different from Control, *p* < 0.05

Oxidative stress is a crucial pathological feature implicated in acute and chronic liver diseases, including drug‐induced liver injury. We next examined the mechanisms by which chronic exposure of hepatocytes to ethanol induced changes in the gene expression. We believed these changes in gene expression occurred due to oxidative stress. Chronic exposure of hepatocytes induced reactive oxygen species. Pretreatment of hepatocytes with *N*‐acetylcysteine (NAC) attenuated ethanol‐induced generation of reactive oxygen species (Figure 8A). We next examined whether the induction of SATB2 by ethanol was due to the generation of ROS (Figure 8B). Chronic exposure of hepatocytes to ethanol induced SATB2 expression. Pretreatment of hepatocytes with NAC (1 mM) attenuated SATB2 expression. These data suggest that the generation of ROS by ethanol is partially responsible for the induction of SATB2.

We next examined whether inhibition of oxidative stress by NAC reversed ethanol‐induced changes in the expression of FASN, GPC3, p53 and FLNB (Figure 8C–F). The expression of FASN, GPC3 and FLNB was induced by ethanol. In contrast, the expression of p53 was inhibited. Pretreatment of hepatocytes with NAC reversed the effects of ethanol on the expression of FASN, GPC3, p53 and FLNB. These data suggest that the generation of oxidative stress in hepatocytes by ethanol is responsible for the changes in gene expression.

## 4 DISCUSSION

Alcohol is a risk factor for HCC, and its effects further accelerate the development of HCC in obesity and diabetes.^45^, ^46^ Here we have demonstrated that chronic exposure of human normal hepatocytes generates oxidative stress and SATB2 gene. SATB2 may be a driving force for developing cancer stem‐like phenotypes in damaged hepatocytes where induction of stem cell markers (CD44, CD90, EpCAM, AFP and LGR5) and pluripotency maintaining factors (POU5F1/Oct4, Sox‐2, Nanog and KLF4) was prominent. SATB2 can directly regulate the expression of Bcl‐2, Nanog, c‐Myc, Klf4 and Oct4 genes by binding to their promoters. Chronic exposure of hepatocytes with ethanol also induced expression of EMT‐related transcription factors (Snail, Slug and Zeb1), N‐Cadherin, and inhibited E‐cadherin expression. The effects of ethanol were exerted through activation of the Wnt/TCF‐LEF1 pathway, which plays a critical role in generating stem cells. Suppression of SATB2 expression in ethanol‐transformed hepatocytes by Crisp/Cas9 technique inhibited markers of stem cells and pluripotency, suggesting an essential role of SATB2 in generating the properties of cancer stem‐like cells. Finally, ethanol induced hepatocellular steatosis, SREBP1 transactional activity, and expression of SREBP1, lipogenic genes (acetyl Co‐A Carboxylase and FASN), inflammatory cytokines (IL‐6, IL‐1β, TNFα), GPC3 and FLNB1, and inhibited p53. *N*‐acetylcysteine reversed the effects of chronic exposure to ethanol in hepatocytes. These data suggest that chronic ethanol exposure of hepatocytes may cause HCC by generating cancer stem cell‐like properties and cellular steatosis.

We hypothesize that exposure of hepatocytes to ethanol generates an inflammatory condition which in turn induces the SATB2 gene. SATB2 is an emerging transcription factor whose biological significance has recently been recognized. It regulates stemness by controlling the expression of pluripotency and self‐renewal factors, and epithelial‐mesenchymal transition.^13^, ^14^, ^15^, ^28^ We have recently demonstrated that the SATB2 gene can induce the transformation of normal epithelial cells into cancer stem cells. Based on gain‐ and loss‐of‐function studies, SATB2 enhances HCC cell proliferation, migration and invasion in vitro and HCC tumorigenicity.^13^ SATB2 might be a potential prognostic marker and a therapeutic target for HCC.^47^, ^48^

Liver CSCs are identified by expressing specific cell surface markers characteristic of stem cell populations, such as CD90, CD44 and EpCAM.^30^, ^49^ EpCAM is a transmembrane glycoprotein that is present in liver stem cells and hepatoblasts.^34^ The expression of EpCAM is associated with cell proliferation and is prominent among CSC‐enriched populations in HCC and many other types of cancers. Moreover, EpCAM expression is associated with cells that exhibit tumour‐initiating capabilities and tumorigenesis. HCC cells expressing EpCAM have more excellent stem cell features, tumour formation and invasion ability than those not expressing EpCAM. These reasons support the use of EpCAM as a target receptor for cancer drug delivery systems. The canonical Wnt/β‐catenin signalling transcriptionally regulates EpCAM expression, inhibiting Wnt/β‐catenin signalling can eliminate EpCAM + cells. Mutations in the β‐catenin gene and aberrant activation of the Wnt/β‐catenin pathway are the most frequently encountered in HCC in the West.

Although alcohol is a risk factor for liver cancer, chronic alcohol use can cause cirrhosis, resulting in the development of liver cancer. As per the reports of the NIAAA, prolonged, heavy drinking has been associated with primary liver cancer. Evidence suggests that the effect of alcohol is modulated by polymorphisms in genes encoding enzymes for EtOH metabolism [e.g. alcohol dehydrogenases (ADH), aldehyde dehydrogenases (ALDH) and cytochrome P450 2E1)], folate metabolism and DNA repair. The mechanisms by which alcohol consumption exerts its carcinogenic effect are not well understood. Several events, including a genotoxic effect of acetaldehyde, increased oestrogen concentration, a role as a solvent for tobacco carcinogens, production of reactive oxygen species and nitrogen species, and changes in folate metabolism have been implicated. Acetaldehyde is the first and most toxic metabolite of alcohol metabolism.^50^, ^51^, ^52^ ADH and ALDH are the main enzymes that regulate alcohol and acetaldehyde's metabolism.^53^ Acetaldehyde reacts with DNA and acts as a carcinogen. In addition, highly reactive, oxygen‐containing molecules (generated during alcohol metabolism) can damage the DNA and induce carcinogenesis.^50^, ^51^, ^52^ A recent study has demonstrated that chronic alcohol intake promotes intestinal tumorigenesis and tumour invasion in genetically susceptible mice, increases in polyp‐associated mast cells, and mast cell‐mediated tumour migration in vitro,^54^ suggesting mast cell‐mediated inflammation could promote carcinogenesis.^54^

Glypican‐3 (GPC3) is a cell surface biomarker that is overexpressed in foetal liver and early‐stage cancer but not in the healthy adult liver.^55^, ^56^ However, in HCC patients, GPC3 is overexpressed at both the gene and protein levels, and its expression predicts a poor prognosis. GPC3 functions in HCC progression by binding to Wnt signalling proteins and growth factors. It plays a significant role in HCC development, angiogenesis and metastasis.^56^ In the present study, ethanol not only induced GPC3 by also activated the Wnt pathway, which plays a role in HCC development.

Ethanol can increase pro‐inflammatory cytokines and trigger an inflammatory response.^57^ On the other hand, ROS promotes the release of proinflammatory cytokines, thereby enhancing the intracellular signal cascade to exacerbate inflammation.^42^, ^58^ One of the important mechanisms of ethanol‐induced liver injury is the production of oxygen free radicals.^59^, ^60^ Excessive production of oxygen free radicals leads to lipid peroxidation. Oxidative stress is activated by fatty acids in cultured human hepatocytes.^61^ In the present study, SATB2 upregulates FASN, an enzyme responsible for lipogenesis, steatosis, NAFLD and HCC.^62^ Hepatic steatosis is directly correlated with FASN expression. N‐Acetyl cysteine (NAC) exhibits antioxidant properties, especially in relation to enhancing endogenous GSH content to counteract oxidative stress. Ethanol induced hepatocellular steatosis, SREBP1 transcription and induced the expression of SREBP1, ACAC, ACLY, GPC3 and FLNB. Inflammatory cytokines (IL‐1β, IL‐6 and TNF‐α) were also induced in ethanol‐treated hepatocytes. Finally, NAC reversed the effects of ethanol on ROS production and induction of FASN, GPC3 and FLNB, suggesting the regulatory role of inflammation and fatty acid synthesis in ethanol‐induced cellular damage.

The expression of p53 is lost in cancer cells while it is highly expressed in normal cells.^43^ p53 loss not only prevents embryonic tumour cells from undergoing oncogene‐induced senescence and apoptosis but also perturbs cell cycle checkpoints.^63^ It has been demonstrated that p53 inhibition by CD44‐enhanced growth factor signalling is required to initiate liver cancer.^64^ In our study, chronic exposure of hepatocytes with ethanol inhibited the expression of p53, suggesting the loss of p53 may give the signal for hepatocyte damage.

In conclusion, our data demonstrate that chronic exposure of hepatocytes with ethanol induces cellular damage in vitro. These damaged cells gained the phenotypes of CSCs (expressed markers of stem cells, EMT, and pluripotency maintain factors) by inducing SATB2 and activating Wnt/TCF‐LEF1 pathway. Further studies are needed to confirm the role of ethanol in hepatocyte transformation and HCC development in vivo.

## CONFLICTS OF INTEREST

The authors have declared that no conflict of interest exists.

## AUTHOR CONTRIBUTIONS

**Wei Yu:** Conceptualization (equal); Data curation (equal); Formal analysis (equal); Investigation (equal); Methodology (equal); Project administration (equal); Validation (equal); Visualization (equal). **Yiming Ma:** Conceptualization (equal); Data curation (equal); Formal analysis (equal); Methodology (equal); Project administration (equal); Validation (equal); Writing – original draft (equal). **Sushant K. Shrivastava:** Methodology (equal); Resources (equal); Writing – review & editing (equal). **Rakesh K. Srivastava:** Resources (equal); Supervision (equal); Writing – review & editing (equal). **Sharmila Shankar:** Resources (equal); Supervision (equal); Validation (equal); Writing – review & editing (equal).

## Data Availability

The data that support the findings of this study are available from the corresponding author upon reasonable request.
